# Direct Laser Writing of Functional QD–Polymer Structure with High Resolution

**DOI:** 10.3390/ma16062456

**Published:** 2023-03-20

**Authors:** Jiubin Jue, Zongsong Gan, Zhijun Luo, Kai Li

**Affiliations:** 1 Wuhan National Laboratory for Optoelectronics, Huazhong University of Science and Technology, Wuhan 430074, China; 2 Key Laboratory of Education Ministry for Information Storage Systems, Huazhong University of Science and Technology, Wuhan 430074, China; 3 Shenzhen Huazhong University of Science and Technology Research Institute, Shenzhen 518057, China

**Keywords:** direct laser writing, functional QD–polymer nanocomposites, feature size and microstructures, highly luminescent structure, absorptive filter

## Abstract

Promising direct laser writing (DLW) technology has been introduced to process functional quantum dot (QD)–polymer nanocomposites. The results reveal that after surface modification, the QDs are compatible with the SR399 monomer, and the homogeneous incorporation of QDs is accordingly obtained owing to the copolymerization and resultant cross-linking of QDs into SR399 resin under DLW processing with a laser wavelength (λ) of 532 nm. Moreover, compared with other scholars, we have proved that the surface modified QDs incorporated into the nanocomposites that can be successfully processed via DLW can reach a concentration of up to 150 mg/mL. Owing to the threshold behavior and nonlinear nature of the DLW process, it is feasible to modify the attendant exposure kinetics and design lines of any small size by selecting an appropriate laser power (*P*) and scan speed (*v*). The superfine feature size of 65 nm (λ/8) of the red QD–polymer suspended line can be tailored by applying the optimized *P* of 15 mW and *v* of 700 μm/s, and the finest green QD–polymer suspended line also reaches 65 nm (λ/8) with the optimized *P* of 14 mW and *v* of 250 μm/s used. Moreover, DLW processed QD–polymer structures present strong and homogeneous photoluminescence emission, which shows great potential for application in high-resolution displays, anti-counterfeit technology, and optical encryption. Additionally, the two types of long pass QD–polymer absorptive filters prepared by DLW exhibit superior optical performance with a considerably high transmittance of more than 90% for red QD–polymer block filter, and over 70% for green QD–polymer block filter in the transmittance region, which means that different filters with specific performance can be easily customized to meet the demand of various microdevices. Therefore, the DLW process can be applied to produce geometrically complex micro- and nanoscale functional structures, which will contribute to the development of advanced optoelectronic devices.

## 1. Introduction

Nanocrystal quantum dots (QDs) are widely promising materials in solar cells, displays, photodetectors, Light Emitting Diodes (LEDs), and fluorescence labels because they exhibit favorable electronic and optical characteristics such as high photoluminescent quantum yield, wide-band absorption and excitation, narrow and size-tunable light emission, and good chemical and photo-stability [1,2,3,4]. However, with the rapid development of nanotechnology and increasing demand for miniature, integrated, and cheap optoelectronic devices with special properties, high-performance functional polymer nanocomposites have attracted much interest. Functional polymer nanocomposites typically consist of polymer resins and other additives at the nanoscale, for example, dyes, particles, and special polymers. Polymer nanocomposites can be fabricated by nanotechniques into two-dimensional (2D) or three-dimensional (3D) micro- and nanoscale structures, which exhibit a series of excellent optical, electrical, and biological properties, thus showing considerable potential applications in advanced optoelectronic devices [5,6,7]. Compared to other nanomaterials, QDs are appreciated for their inherent merits. Because of the quantum confinement effect, the absorption and emission spectra of QDs can be continuously and finely tuned over wavelengths ranging from deep ultraviolet to mid-infrared simply by changing the size, shape, and composition of the QDs. Therefore, functional QD–polymer nanocomposites are ideal materials for achieving a highly luminescent and broadband absorptive filter microstructure [8,9,10]. Conventional processing methods, such as vacuum deposition and photolithography, face some challenges in the fabrication of polymer nanocomposites, including high vacuum environments, expensive customized masks, and complicated and time-costly procedures. In addition, they are unable to flexibly fabricate complex 3D structures, thereby limiting further applications of polymer nanocomposites significantly [11,12]. Considering the above points, it is essential to develop a novel processing method for the fabrication of complex-shaped QD–polymer nanocomposites with high resolution.

In recent decades, with the advancement of additive manufacturing technology, increasing attention has been focused on the application of direct laser writing (DLW) additive manufacturing technology in the net-shape fabrication of geometrically complex components [13,14,15,16]. As a powerful laser-based additive manufacturing technology, DLW, which is based on a nonlinear multi-photon absorption and polymerization mechanism, can be used to manufacture complex nanostructures by selectively polymerizing the polymer resin line by line and then layer by layer with a tightly focused laser beam according to computer-aided design (CAD) models of the structures. The unique processing manner and processing mechanisms of DLW offer high potential for manufacturing a wide range of high-performance complex shaped three-dimensional semiconductors, metals, polymers, and polymer nanocomposites at low-cost, short-cycle, and without any dedicated mask [17,18,19,20]. More importantly, DLW is characterized by high peak power and nonlinear absorption. This contributes greatly to producing a fairly tiny polymerization region at the focal spot and finally acquiring super-high spatial resolution below the diffraction limit. Moreover, the DLW process has a great advantage in the tailoring and integration of functionally advanced nanodevices with a designed configuration. DLW has been demonstrated to be successful in fabricating various micro- and nanodevices for many applications [21,22,23,24,25,26,27]. Therefore, the introduction of DLW technology in the fabrication of QD–polymer nanocomposites is an effective choice to compensate for the existing deficiencies of conventional methods.

Nonetheless, studies on the manufacturing of QD–polymer nanocomposites as highly luminescent microstructures and absorptive filter microstructures using 532 nm DLW technology are rather limited. Some researchers report the fabrication of luminescent microstructures by DLW of processable QD–polymer nanocomposites, but the doping concentration of ODs is relatively low, usually 5 mg/mL to 15 mg/mL. In this study, QDs of different sizes first undergo surface modification and are then manufactured using 532 nm DLW at different laser processing parameters. This study aims to significantly increase the doping concentration of QDs and simultaneously investigate the feature size, microstructure development, and optical performance of QD–polymer nanocomposites fabricated using 532 nm DLW. In addition, the influence of the DLW processing parameters on the feature size, and microstructure characteristics is studied in detail and the underlying physical and chemical metallurgical mechanisms accounting for the variation are proposed. Simultaneously, highly luminescent structures and two types of long pass absorptive filters are present.

## 2. Materials and Methods

### 2.1. Materials

Synthesis of quantum dots: The original oleic acid (OA) capped red CdSe/CdZnS/ZnS QDs and green CdSe/ZnS QDs were synthesized according to a previously reported method [28,29].

Surface modification of quantum dots: 5 mg OA capped red CdSe/CdZnS/ZnS QDs or green CdSe/ZnS QDs and 30 mg β-carboxyethyl acrylates (CEAs) were added to 5 mL cyclohexane, and stirred at room temperature for 10 h. After stirring, the CEA surface modified QDs precipitated completely in cyclohexane and were further separated via centrifugation at 10,000× *g* rpm. Subsequently, cyclohexane was poured out and the CEA surface modified QDs were purified via washing with ethanol three times to remove the remaining CEAs.

Functional QD–polymer nanocomposites: The 5 mg surface modified QDs were first dissolved in 3 mL dichloromethane, which was then mixed with 975 mg dipentaerythritol pentaacrylate (SR399) resin monomer and 5 mg of the highly efficient IRG819 photoinitiator. Next, the mixtures were slowly stirred, heated, and kept at 40 °C to evaporate the whole dichloromethane. Finally, to remove the bubbles, a vacuum pumping experiment of the mixture was carried out. Through the above steps, functional red QD–polymer nanocomposites containing 0.5% IRG819 photoinitiator and 5 mg/mL red CdSe/CdZnS/ZnS QDs, and functionalized green QD–polymer nanocomposites comprising 0.5% IRG819 photoinitiator and 5 mg/mL green CdSe/ZnS QDs were successfully prepared.

### 2.2. DLW Process

**Figure 1:**
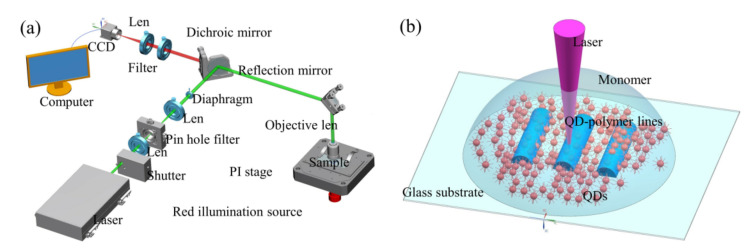
(**a**) Schematic diagram of the DLW apparatus and (**b**) the generation process of the QD–polymer composites.

As schematically depicted in Figure 1a, the homemade DLW system mainly consisted of a 532 nm femtosecond (fs) laser at a repetition rate of 80 MHz with a 140 fs pulse width, a mechanical shutter, a 3D PI stage, a red illumination source, a CCD monitoring system, and a computer system for process control. Prior to the shaping process, a drop of the QD–polymer nanocomposites was sealed into two pieces of cover glass with a gap of 50 μm. The fs laser then experienced beam expansion and was tightly focused through a high numerical aperture oil objective (100×, NA = 1.4, Olympus, Tokyo, Japan). DLW is a multi-photon polymerization technique based on a photochemical process in which the tightly focused femtosecond laser with a high energy then irradiates deeply into the volume of QD–polymer nanocomposites. It subsequently induces very specific chemical changes and polymerization of small unsaturated molecules in the liquid state to solid macromolecules [14,30,31]. Subsequently, according to the CAD data of the structure, the high energy laser scanned the QD–polymer nanocomposites to form a layerwise profile in a simple linear raster scan pattern by controlling the motion of a PI stage (Physik Instrumente P-563) and mechanical shutter. In this way, arbitrary three-dimensional structures can be realized by the motion of the laser beam line by line and then layer by layer (Figure 1b). After fabrication, the coverslip containing the structure was rinsed in pure acetone for 5 min, and then in pure isopropanol for 2 min.

The resolution of the DLW fabrication, which is expressed by the voxel or width of the line, is of significant importance for microdevices with different requirements. In the past decades, many scholars have studied the related influencing factors of controllable resolution using a large number of experimental and theoretical analyses [17,22,32,33]. This indicates that the fabrication resolution is not only affected by the technical equipment system, but also depends on the material properties and laser processing parameters. These factors simultaneously function in the DLW process and influence each other. For a given material formulation and an optimized technical equipment system, the feature size of polymerized structures is demonstrated to be extremely sensitive to laser processing parameters, such as *P* or *v*. In this study, a 532 nm DLW based on two-photo absorption is a third-order nonlinear optical process. The rate of laser absorption is proportional to the square of the laser intensity. A threshold behavior also exists during the DLW process. Studies by many scholars have shown that under the combined effect of the nonlinear nature and threshold behavior, it is feasible to modify the attendant exposure kinetics and design voxels of any small size by choosing an appropriate *P* and *v*. More importantly, a super-high resolution well below the diffraction limit can be obtained when the applied *P* is adjusted to approach the polymerized threshold value [17,34,35]. Therefore, in the present study, different laser processing parameters were applied to investigate their influence on the resolution. The applied *P* was set to 14, 15, 18, 22, 26, 27, 31, and 33 mW using the DLW control program. The corresponding *v* was changed from 100 to 700 μm/s at intervals of 50 μm/s.

### 2.3. Microstructural Characterization and Optical Properties Tests

UV-Vis absorption and PL emission spectra were measured in the range of 400–700 nm using a A590 UV-vis spectrometer (AOE, Shanghai, China) and PL Aurora 4000 fluorescence spectrophotometer (Qingxuan, Changchun, China), respectively. The morphological features and size of the suspended lines were revealed using a Hitachi S-4800 field emission scanning electron microscope (FE-SEM, Tokyo, Japan) at 2 kV and 3 kV. The microstructures of the QDs before and after DLW polymerization were analyzed using an FEI Tecnai G2 20 transmission electron microscope (Hillsboro, OR, USA) with an accelerating voltage of 200 kV. The PL emission spectra of the DLW processed QD–polymer structure were collected using a LabRAM HR800 laser microscope confocal Raman spectrometer (HORIBA Jobin Yvon, Paris, France). Fluorescence images were obtained using an OLYMPUS-BX51 fluorescence microscope. The transmission spectra of the DLW processed long pass absorptive filters were determined using a microscopic infrared spectrometer.

## 3. Results

**Figure 2:**
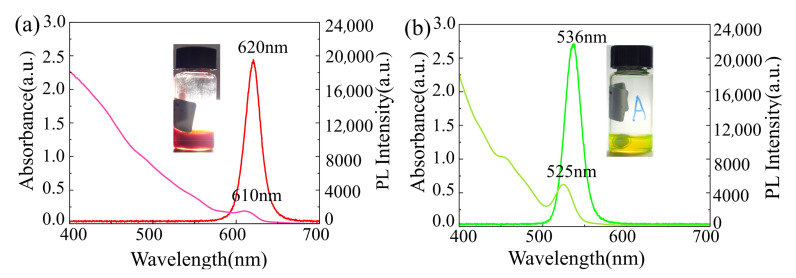
UV-Vis absorption and PL emission spectra of functional quantum dots (inset: photograph of functional QDs dispersed in SR399 monomer observed under visible light): (**a**) red CdSe/CdZnS/ZnS QDs; (**b**) green CdSe/ZnS QDs.

It is well known that the optical properties of QD–polymer nanocomposites strongly depend on the size of QDs, the uniformity of QDs, and the interfacial bonding behavior of the QDs/matrix [36,37]. QDs are generally composed of inorganic nanoparticles that are surrounded by a layer of organic ligands. The organic ligands determine the surface chemistry of the QDs and hence play an important role in their processing, functionalization, and incorporation into various matrices. Nevertheless, because of the interdigitation of stabilizing ligand alkyl terminals, QDs synthesized by conventional methods aggregate and segregate into large clusters, thus resulting in a considerably heterogeneous distribution of QDs and a poor QD/matrix interface. As a result, the optical properties of QD–polymer nanocomposites are severely degraded. Surface modification of QDs with special ligands is considered an effective method to address the above problems [38,39]. In this study, a special CEA with a reactive acrylic double bond was selected as the exchanger; hence, it is easy to copolymerize with acrylic monomers in lotion polymerization and radiation curing systems. After the ligands of the OA capped red CdSe/CdZnS/ZnS QDs or green CdSe/ZnS QDs were exchanged with CEA, the surfaces of the QDs contained polymerizable ligands, which had a chemical composition similar to that of the SR399 resin, thus preventing aggregation of QDs and leading to the homogeneous incorporation of QDs in the SR399 polymer resin. Moreover, it enables the successful fabrication of 3D microstructures without laser absorption and scattering arising from QD aggregation. The UV-Vis absorption and PL emission spectra of surface modified QDs under 365 nm UV light and QDs dispersed in SR399 resin observed under visible light are shown in Figure 2. The surface modified red CdSe/CdZnS/ZnS QDs and green CdSe/ZnS QDs exhibited broad absorption bands and strong PL emission with distinguishable absorption bands at 610 nm and 525 nm, respectively. For the red CdSe/CdZnS/ZnS QDs, the maximum emission peak was at 620 nm with full-width at half-maximum (FWHM) values of 22 nm, whereas the green CdSe/ZnS QDs exhibited a maximum emission peak value of 536 nm and FWHM value of 22 nm, which indicated that the particle size of the QDs was relatively narrow and monodisperse. From the inset in Figure 2, it was clear that when surface modified QDs were added to the SR399 monomer, they were well dispersed in the SR399 monomer with reddish and yellow-greenish colors. This confirmed that surface modified QDs with polymerizable ligands were compatible with the SR399 monomer, resulting in a uniformly dispersed state that was suitable for the DLW process.

**Figure 3:**
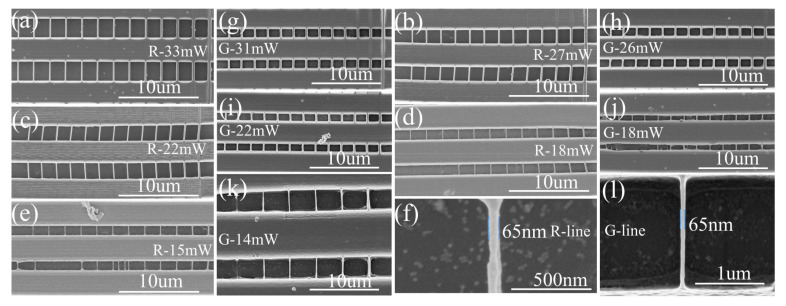
FE-SEM images illustrating the morphologies of the DLW processed 5 mg/mL red QD–polymer suspended lines (**a**–**e**): (**a**) 33 mW; (**b**) 27 mW; (**c**) 22 mW; (**d**) 18 mW; (**e**) 15 mw; and 5 mg/mL green QD–polymer suspended lines (**g**–**k**): (**g**) 31 mW; (**h**) 26 mW; (**i**) 22 mW; (**j**) 18 mW; (**k**) 14 mw, and SEM image of the smallest suspended line of the DLW processed red QD–polymer composites (**f**) and green QD–polymer composites (**l**). R stands for red QD–polymer and G stands for green QD–polymer in the figure. For each laser power, the suspended lines from right to left were fabricated using *v* from 100 to 700 μm/s with an interval of 50 μm/s.

**Figure 4:**
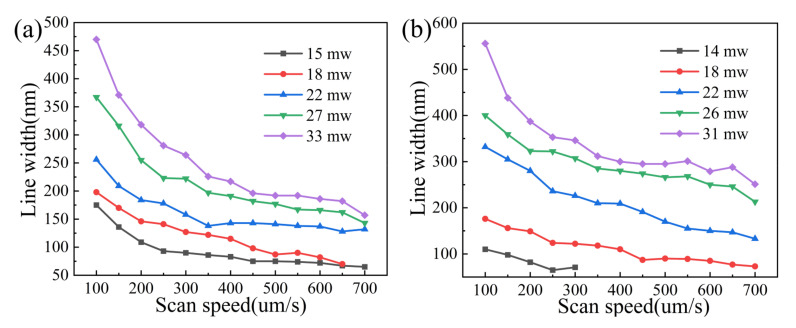
The effect of laser processing parameters on the width of the DLW processed red QD–polymer suspended lines (**a**) and green QD–polymer suspended lines (**b**).

SEM images illustrating the morphologies of DLW processed 5 mg/mL red QD–polymer suspended lines and 5 mg/mL green QD–polymer suspended lines at various laser processing parameters are shown in Figure 3, while the effect of laser processing parameters on the width of the suspended lines is illustrated in Figure 4. Nanolines below 100 nm are known to be easily washed out during development or ablated by the electronic beams of SEM. In this study, the suspending bridge method was employed to observe them clearly. A series of suspended lines was fabricated on the surfaces of two parallel rectangular supports. The rectangular supports were polymerized with a high *P* of 30 mw and a low *v* of 150 μm/s with the purpose of having enough bonding strength. Instead, to investigate the variation in the width of the lines as a function of laser processing parameters, the suspended lines were achieved at various *P* values of 15 mW, 18 mW, 22 mW, 27 mW, 33 mW for red QD–polymer nanocomposites and 14 mW, 18 mW, 22 mW, 26 mW, 31 mW for green QD–polymer nanocomposites. For each laser power, the suspended lines were fabricated using *v* from 100 to 700 μm/s with an interval of 50 μm/s. In the DLW process, it is established that a too low laser power is unable to polymerize the monomer, but when the laser power is too high, greater than a particular value, laser-induced breakdown occurs, which boils the resin and causes intense damage, thus preventing the formation of lines. Therefore, appropriate laser processing windows were selected in this study.

From Figure 3, all suspended lines were found to be smooth, continuous, and complete. The width of the suspended lines represented the feature size. It was obvious that both *P* and *v* played a significant role in affecting the feature size of the DLW processed QD–polymer suspended lines. The widths of the suspended lines showed a trend of continuous increase with increasing *P*. Simultaneously, the higher *v* was, the finer the suspended lines became. In the case of DLW processed red QD–polymer suspended lines, with decreasing applied *P*, the width of the suspended lines decreased from 470, 367, 256, 198 to 175 nm at a given *v* of 100 μm/s. The width decreased from 157, 143, 132 to 65 nm at a constant *v* of 700 μm/s when used *P* decreased from 33, 27, 22 to 15 mW (Figure 3a–e and Figure 4a). As for DLW processed green QD–polymer suspended lines, when the used *P* decreased from 31, 26, 22 to 18 mW, the width of the suspended lines experienced a continuous decline: 556, 400, 332 to 176 nm at a given *v* of 100 μm/s, which decreased from 251, 213, 133 and 73 nm at a constant *v* of 700 μm/s (Figure 3g–j and Figure 4b). Upon decreasing the applied *P* to 14 mW, the width of the suspended lines at the *v* of 100 μm/s and 250 μm/s further decreased to 110 nm and 65 nm (Figure 3k and Figure 4b), respectively. The above results revealed that a significant refinement of the red QD–polymer suspended lines occurred, and the finest suspended line with a width of 65 nm was generated with the optimized *P* of 15 mW and *v* of 700 μm/s applied. The feature size of the green QD–polymer suspended lines underwent a great degree of refinement, which also reached 65 nm with the optimized *P* of 14 mW and *v* of 250 μm/s used. Therefore, it was considered that the feature size of DLW processed QD–polymer suspended lines could be reduced to 65 nm (λ/8) by optimizing the applied *P* and *v*.

In this study, when the high energy laser irradiated the QD–polymer composites, they absorbed energy in an extremely short time. When a relatively low *P* of 14 mW, 15 mW, and a constant *v* was used, the input laser energy was low. In addition, owing to the Gaussian distribution of the laser beam, the energy gradient between the center and edge of the active region was extremely steep. As stated above, the rate of two-photo absorption was the square of the laser intensity dependence. Consequently, only the center of the active region could absorb the laser energy well for polymerization, which led to a significantly refined resolution. Polymerization may not occur at the edge of the active region owing to insufficient laser intensity (Figure 3e,k). When the employed *P* was increased from 18 mW to 33 mW, there was a larger area of the light distribution with sufficient power to induce polymerization, thus significantly improving the polymerization degree. In this case, in addition to the center, the increasing number of regions at the edge of the active region qualified for polymerization, thus producing a comparatively coarse fabrication resolution (Figure 3a–d,g–j). Furthermore, from Figure 3 and Figure 4, at a given *P*, when a high *v* of 700 μm/s was used, the laser–material interaction time was extremely short, and there was very little energy accumulated, thus limiting the generation and diffusion of the active radicals to a large degree. This facilitated the resolution improvement. Decreasing *v* from 650 to 100 μm/s prolonged the laser–material interaction time. Thus, the active region absorbed more laser energy. In this case, more active radicals were generated and diffused from the center to the surrounding space owing to the chemical concentration gradient, thereby decreasing the acquired fabrication resolution. It is worth mentioning that a superfine feature size of 65 nm (λ/8) could be tailored by selecting a relatively low *P* and high *v*.

**Figure 5:**
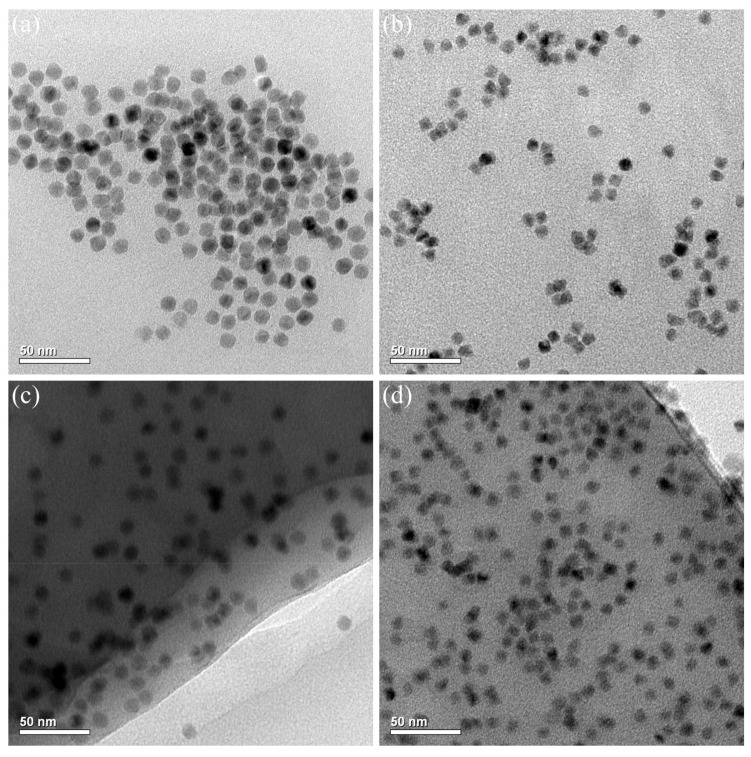
TEM images illustrating the morphologies and distribution state of red QDs (**a**,**c**) and green QDs (**b**,**d**) before DLW polymerization (**a**,**b**), and after DLW polymerization (**c**,**d**) of QD–polymer composites with QD doping concentrations of 5 mg/mL using the applied *P* of 33 mW for (**c**) and 31 mW for (**d**) at the *v* of 100 μm/s.

Figure 5 depicts transmission electron microscopy (TEM) images illustrating the morphologies and distribution state of red QDs and green QDs before DLW polymerization and after DLW polymerization of QD–polymer composites with QDs doping concentration of 5 mg/mL using the applied *P* of 33 mW for (c) and 31mW for (d) at the *v* of 100 μm/s. To perform TEM characterization, the red QDs or green QDs were dissolved in the dichloromethane, which was then dropped onto a TEM grid and heated at 40 °C to evaporate the dichloromethane. It was clearly observed that the red QDs exhibited a spherical morphology with an average particle size of 10.3 nm (Figure 5a). Green QDs with a spherical structure and a mean particle size of 8.4 nm were observed (Figure 5b). They all showed an extremely narrow particle size distribution in a monodisperse state. Furthermore, advanced DLW technology has been introduced to fabricate QD–polymer nanocomposites. As shown in Figure 5c,d, the ODs did not show any change in size and morphology after polymerization. In addition, their distribution was considerably homogeneous in the polymerized SR399 resin. It can be concluded that even if the DLW processed QD–polymer suspended lines had a size of tens of nanometers, they also contained sufficient QDs inside, thus showing excellent optical properties. In this study, the original QDs were surface modified with functional and photosensitive groups, which were compatible with SR399 resin. Consequently, during the 532 nm DLW process, the QDs and the SR399 resin were triggered and polymerized together, thus resulting in the cross-linking of ODs into an interconnected SR399 resin matrix. Under these conditions, uniform incorporation of the surface-modified QDs was finally obtained and a compatible interface between them was established.

**Figure 6:**
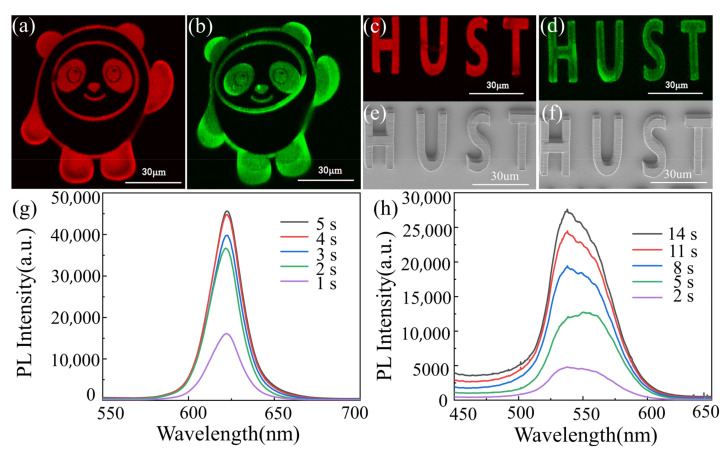
The fluorescence images of the DLW processed 5 mg/mL red QD–polymer microstructure of 2D bingdundun, 3D hust (**a**,**c**), as well as 5 mg/mL green QD–polymer microstructure of 2D bingdundun, 3D hust (**b**,**d**); FE-SEM images of the DLW processed 5 mg/mL red QD–polymer 3D hust (**e**) and 5 mg/mL green QD–polymer 3D hust (**f**); PL emission spectra of the DLW processed 5 mg/mL red QD–polymer microstructure (**g**) and 5 mg/mL green QD–polymer microstructure (**h**) with an excitation wavelength of 325 nm at various exposure times.

The fluorescence images of the DLW processed 5 mg/mL red QD–polymer microstructure of 2D bingdundun, 3D hust, as well as the 5 mg/mL green QD–polymer microstructure of 2D bingdundun, 3D hust, FE-SEM images of the DLW processed 5 mg/mL red QD–polymer 3D hust and 5 mg/mL green QD–polymer 3D hust, and PL emission spectra of the DLW processed 5 mg/mL red QD–polymer microstructure and 5 mg/mL green QD–polymer microstructure at various exposure times are depicted in Figure 6. As shown in Figure 6, highly luminescent 2D and 3D structures were successfully obtained by DLW processing of QD–polymer nanocomposites, which showed strong and homogeneous PL emission at an excitation wavelength of 325 nm. This indicated that the surface modified QDs were chemically stable and retained their optical characteristics after laser irradiation. Moreover, the 2D and 3D structures exhibited good integrity, confirming that the functional QD–polymer nanocomposites were suitable for DLW processing. As for the PL emission spectra, an excitation wavelength of 325 nm was used, and the excitation power was set at 0.3 mW with different exposure times. Two emission peaks at approximately 620 and 536 nm were clearly observed, which corresponded to the emission of surface modified red and green QDs, respectively. Additionally, the emission intensity increased when the exposure time was prolonged, because more QDs trapped in the polymer matrix were excited. In view of the above analysis, it was considered that fluorescent structures of different colors or even microdevices could be acquired using DLW technology by incorporating various functional QDs into the basic resin.

**Figure 7:**
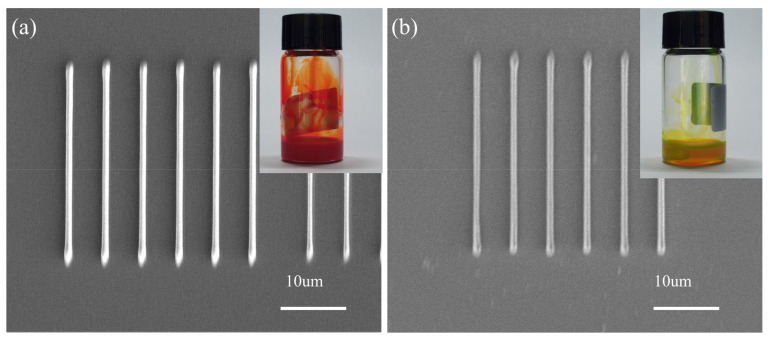
FE-SEM images illustrating the morphologies of the DLW processed 150 mg/mL red QD–polymer lines (**a**), 150 mg/mL green QD–polymer lines (**b**) at a *P* of 35 mW and a *v* of 30 μm/s, and 150 mg/mL QD–polymer nanocomposites in the illustration.

Currently, most studies report microfabrication of processable QD–polymer nanocomposites by DLW, which contain QDs with low quantities, usually 5 mg/mL to 15 mg/mL [12,18,24,36]. QD–polymer nanocomposites incorporating a high concentration of QDs are not suitable for DLW processing. This is not conducive to the improvement of the performance of microdevices. To deal with the problems, in this study, a special CEA with a reactive acrylic double bond was selected as the exchanger. The original QDs were surface modified with functional and photosensitive groups similar to that of the SR399 resin. Consequently, during the 532 nm DLW process, the QDs and the SR399 resin were triggered and polymerized together, thus resulting in the cross-linking of ODs into an interconnected SR399 resin matrix. The above factors provided the possibility of incorporation of high concentration of QDs into the polymer, which thus can be suitable for DLW processing. In this study, we prepared functional red QD–polymer nanocomposites containing 150 mg/mL red CdSe/CdZnS/ZnS QDs, and functionalized green QD–polymer nanocomposites comprising 150 mg/mL green CdSe/ZnS QDs. Figure 7 shows the DLW processed 150 mg/mL red QD–polymer lines, 150 mg/mL green QD–polymer lines at a *P* of 35 mW and a *v* of 30 μm/s, and 150 mg/mL QD–polymer nanocomposites in the illustration. It was shown that the DLW processed lines were smooth and complete without fracture. This proved that the surface modified QD–polymer nanocomposites with high concentration QDs can be used for DLW processing.

The filters can absorb or weaken the light waves outside the required wavelength range, and simultaneously transmit the light of the target wavelength, which has important applications in many fields of detection, remote sensing, medical treatment, solid-state lighting, display, and hyperspectral imaging [40,41]. With the development and progress of technological levels, higher requirements have been proposed for the research and application of optical filters, among which miniature, high-integration, and high-resolution optical filters with multiple-channels are the future direction. In recent years, QDs have received extensive research attention owing to their unique properties, such as size-dependent tunable band gaps. In addition, the light absorption property, which is one of the most stable properties of QDs, can be continuously and finely tuned over wavelengths ranging from deep ultraviolet to mid-infrared, simply by changing the size, shape, and composition of the QDs. This tunability makes it possible to realize a spectral device containing many different filter channels more easily and economically, which indicates that QDs have practical feasibility as ideal filter materials for tunable absorption. In addition, DLW has demonstrated a huge advantage in the fabrication of arbitrary complex structures with high resolution. Based on the above ideas, this study combines the merits of DLW and the tunable absorption properties of QDs to prepare absorptive filters and further study their properties.

**Figure 8:**
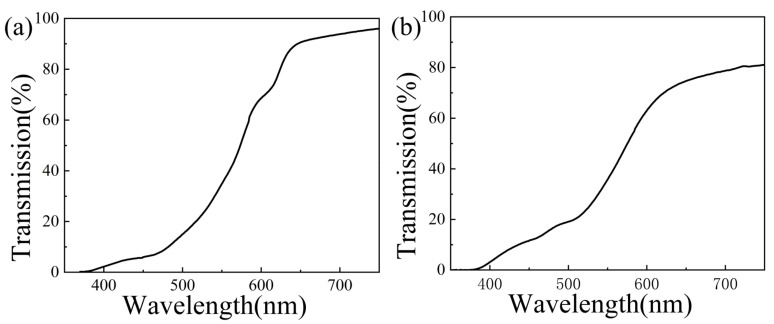
The transmittance spectra of the DLW processed 150 mg/mL red QD–polymer block filter (**a**) and 150 mg/mL green QD–polymer block filter (**b**) at a *P* of 35 mW and a *v* of 30 μm/s.

The fluorescent emissions of red and green QDs were first quenched by adding a small amount of p-phenylenediamine to the functional 150 mg/mL QD–polymer nanocomposites to prevent artifacts produced by the emitted light from the QDs, which was subsequently accompanied by the DLW processing at a *P* of 35 mW and a *v* of 30 μm/s. The transmittance spectra of the DLW processed red QD–polymer block filter and green QD–polymer block filter are shown in Figure 8. It was observed that two types of long pass absorptive filter were acquired. The cut-off band was at 400–517 nm for the DLW processed red QD–polymer block filter, which had an absorption peak at 610 nm. Simultaneously, a maximum transmittance peak of 20% and average transmittance of 8.4% were observed in the cut-off region. The corresponding transmittance region at 646–700 nm presented a stable and high transmittance of more than 90%. The transition region was between them (Figure 8a). However, the DLW processed green QD–polymer block filter, whose absorption peak was located at 525 nm, had a cut-off band of 400–507 nm. The maximum transmittance peak in the cut-off region was approximately 20% at 507 nm, and the average transmittance was 12%. Instead, the transition region was at 507–622 nm. The transmittance region was at 622–700 nm, and the transmittance exceeded 70% (Figure 8b). In summary, long pass QD absorptive filters fabricated via DLW showed outstanding optical performance with an extremely good cut-off characteristic in the cut-off region and a considerably high transmittance in the transmittance region, which is comparable to that of the QD absorptive filters prepared by Jie Bao et al. [9]. Therefore, different filters with specific performance can be easily customized to meet the demands of various microdevices.

## 4. Conclusions

The study reported the spatial resolution, microstructure development, and optical performance of functional QD–polymer nanocomposites fabricated via DLW. The following conclusions could be reached: the surface modification of QDs with polymerizable ligands could prevent the aggregation of QDs, thus leading to the homogeneous incorporation of them in the SR399 polymer matrix and a significant increase in the concentration of QDs incorporated into the QD–polymer nanocomposites. The doping concentration of QDs can reach up to 150 mg/mL, which paved the way for the development of high-performance microdevices fabricated via DLW technology. Both *P* and *v* significantly affected the feature size of the DLW processed QD–polymer suspended lines. The widths of the DLW processed red QD–polymer suspended lines and green QD–polymer suspended lines showed a trend of continuous increase with increasing *P*. Simultaneously, the higher *v* was, the smaller the suspended lines became. Owing to the threshold behavior and nonlinear nature of the DLW process, the superfine feature size of 65 nm (λ/8) of the red QD–polymer suspended line could be tailored by applying the optimized *P* of 15 mW and *v* of 700 μm/s, and the finest green QD–polymer suspended line also reached 65 nm (λ/8) with the optimized *P* of 14 mW and *v* of 250 μm/s used. DLW processed 2D and 3D structures presented strong and homogeneous PL emission, which showed great potential for application in high-resolution displays, anti-counterfeit technology, optical encryption, and optical information storage. Additionally, the two types of long pass QD–polymer absorptive filters prepared via DLW exhibit superior optical performance with a considerably high transmittance of more than 90% for red QD–polymer block filter, and over 70% for green QD–polymer block filter in the transmittance region, which means that different filters with specific performance can be easily customized to meet the demand of various microdevices. Therefore, the DLW process can be applied to produce geometrically complex micro- and nanoscale functional structures, which will contribute to the development of advanced optoelectronic devices.

## Data Availability

The data presented in this study are available on request from the corresponding author.
